# AAV-Mediated Gene Delivery in a Feline Model of Sandhoff Disease Corrects Lysosomal Storage in the Central Nervous System

**DOI:** 10.1177/1759091415569908

**Published:** 2015-04-01

**Authors:** Hannah E. Rockwell, Victoria J. McCurdy, Samuel C. Eaton, Diane U. Wilson, Aime K. Johnson, Ashley N. Randle, Allison M. Bradbury, Heather L. Gray-Edwards, Henry J. Baker, Judith A. Hudson, Nancy R. Cox, Miguel Sena-Esteves, Thomas N. Seyfried, Douglas R. Martin

**Affiliations:** 1 Boston College Biology Department, Chestnut Hill, MA, USA; 2 Scott-Ritchey Research Center, College of Veterinary Medicine, Auburn University, AL, USA; 3 Department of Anatomy, Physiology & Pharmacology, College of Veterinary Medicine, Auburn University, AL, USA; 4 Department of Clinical Sciences, College of Veterinary Medicine, Auburn University, AL, USA; 5 Department of Pathobiology, College of Veterinary Medicine, Auburn University, AL, USA; 6 Department of Neurology and Gene Therapy Center, University of Massachusetts Medical School, Worcester, MA, USA; Victoria J. McCurdy is now at Department of Biological Sciences, Mississippi State University, Mississippi, USA; Allison M. Bradbury is now at Department of Clinical Studies, University of Pennsylvania School of Veterinary Medicine, Philadelphia, Pennsylvania, USA

**Keywords:** adeno-associated virus, β-hexosaminidase, ganglioside, gene therapy, Sandhoff disease

## Abstract

Sandhoff disease (SD) is an autosomal recessive neurodegenerative disease caused by a mutation in the gene for the β-subunit of β-N-acetylhexosaminidase (Hex), resulting in the inability to catabolize ganglioside GM2 within the lysosomes. SD presents with an accumulation of GM2 and its asialo derivative GA2, primarily in the central nervous system. Myelin-enriched glycolipids, cerebrosides and sulfatides, are also decreased in SD corresponding with dysmyelination. At present, no treatment exists for SD. Previous studies have shown the therapeutic benefit of adeno-associated virus (AAV) vector-mediated gene therapy in the treatment of SD in murine and feline models. In this study, we treated presymptomatic SD cats with AAVrh8 vectors expressing feline Hex in the thalamus combined with intracerebroventricular (Thal/ICV) injections. Treated animals showed clearly improved neurologic function and quality of life, manifested in part by prevention or attenuation of whole-body tremors characteristic of untreated animals. Hex activity was significantly elevated, whereas storage of GM2 and GA2 was significantly decreased in tissue samples taken from the cortex, cerebellum, thalamus, and cervical spinal cord. Treatment also increased levels of myelin-enriched cerebrosides and sulfatides in the cortex and thalamus. This study demonstrates the therapeutic potential of AAV for feline SD and suggests a similar potential for human SD patients.

## Introduction

Sandhoff disease (SD) is a progressive neurodegenerative disorder caused by a deficiency of *β*-N-acetylhexosaminidase (Hex, EC 3.2.1.52; Sandhoff et al., 1968). Hex comprises two subunits, *α* and *β*, that dimerize to form separate isozymes: HexA (*α* *β*) and HexB (*β* *β*). Mutations in the gene for the *β*-subunit of Hex cause SD, leading to excessive accumulation of the ganglioside GM2 and its asialo derivative GA2 within lysosomes (Gravel et al., 1995). Disease onset ranges from infancy to adulthood and is marked by progressive neurodegeneration, brain dysfunction, and death. In addition to ganglioside accumulation, SD is also characterized by a reduction in myelin-enriched glycolipids, cerebrosides and sulfatides (Sandhoff et al., 1971; Denny et al., 2006; Baek et al., 2008).

Although the cause of SD has been known for decades, the disease remains incurable today. Several experimental therapies have been examined for the treatment of SD including bone marrow transplantation, stem cell therapy, substrate reduction therapy, caloric restriction, enzyme replacement therapy, and gene therapy (Rattazzi et al., 1982; Norflus et al., 1998; Jeyakumar et al., 1999; Cachon-Gonzalez et al., 2006; Denny et al., 2006; Lee et al., 2007; Arthur et al., 2013; Bradbury et al., 2013). Recent studies revealing the success of adeno-associated virus (AAV) gene therapy in treating murine and feline SD models demonstrate the potential for successful treatment of human GM2 gangliosidosis (Sargeant et al., 2011; Cachon-Gonzalez et al., 2012; Bradbury et al., 2013).

AAV gene therapy in SD mice resulted in survival to 2 years of age, compared with approximately four months of age for untreated SD mice (Cachon-Gonzalez et al., 2006, 2012), which prompted testing in SD cats. The naturally occurring feline model of SD has been maintained as a research colony and thoroughly characterized for more than 35 years (Cork et al., 1977, 1978; Martin et al., 2004; Baek et al., 2009; Bradbury et al., 2009). The cat brain is >50 times larger than a mouse brain and is more complex. Also, human anatomy and lipid profiles are more similar to cat than mouse; thus the treatment of cats was chosen to act as a bridge between mouse experiments and human clinical trials (Baek et al., 2009; Bradbury et al., 2013).

After bilateral injection of the thalamus alone, SD cats treated with AAV1 vectors expressing human Hex had reduced GM2 ganglioside storage and an increased lifespan (Bradbury et al., 2013). However, a pronounced humoral immune response was detected in treated cats, and restoration of Hex activity to the cerebellum was minimal. Second-generation vectors incorporating the AAVrh8 capsid and feline Hex led to improved longevity with minimal immune response (Bradbury et al., 2013). Here, we optimize therapy of the cerebellum and spinal cord by adding intracerebroventricular (ICV) injection of AAVrh8 vectors to the thalamic delivery route proven effective at treating the diencephalon. Also, we examine the lipid profiles of AAVrh8-treated SD cats to better assess therapy distribution throughout the central nervous system (CNS) and feasibility of this approach for human clinical application.

## Materials and Methods

### Animal Surgery and Treatment Groups

The breeding colony of SD cats was maintained at Auburn University, AL. The institutional animal care and use committee approved the research described herein. Affected SD cats were treated at 1.1 to 1.6 months of age (disease onset, 1.3 ± 0.2 months). Anesthesia was induced with ketamine (10 mg/kg) and dexmedotomidine (0.04 mg/kg) through an intravenous catheter and maintained using isoflurane (0.5% to 1.5%) in oxygen delivered through an endotracheal tube. Cats were positioned sternally for intracranial injection using a Horsley–Clark stereotaxic apparatus (David Kopf Instruments, Tujunga, CA) and injected with vector bilaterally in the thalamus and in the left lateral ventricle. Craniotomy sites were made with a 20-gauge needle at the following distances (in cm) from bony landmarks on the skull:thalamus (relative to bregma), anterior-posterior (AP)−0.65, mediolateral (ML) ±0.4, dorsoventral (DV) −1.6 (from meninges); left lateral ventricle, caudo-thalamic notch located by ultrasound guidance through the bregma or median suture after a 1.5-cm incision of the scalp. Vector was delivered using a Hamilton syringe (Harvard Apparatus, Holliston, MA) with a noncoring needle (22-25 G). A total of 70 μl was injected into each thalamus in 10 to 20 μl boluses. Injection rate was 2 μl/min, and the needle was raised 0.15  cm between boluses. A total of 200 μl was injected into the left lateral ventricle at a rate of 15 μl/min. The total vector dose was 1.1 × 10^12^ vector genomes, which comprise a 1:1 ratio of AAVrh8 vectors expressing the feline Hex *α* or *β* subunit, described previously (Bradbury et al., 2013). Vectors included the hybrid chicken *β*-actin promoter (Cytomegalovirus immediate-early enhancer fused to the chicken *β*-actin promoter; Matalon et al., 2003) and woodchuck hepatitis posttranscriptional regulatory element (WPRE). AAV-treated cats (*n* = 4) were euthanized 16 weeks postinjection for biochemical analysis of therapeutic effect. Controls were age-matched untreated normal cats (*n* = 4 for all analyses) and untreated SD cats (*n* = 5 for enzyme analysis, *n* = 6–7 for storage analysis, and *n* = 14 for clinical analysis). Disease progression was scored on a 10-point scale based on gait defects, compiled from untreated SD cats over the last 5 years. Cats received the following scores based on gait. Age of onset ± standard deviation are shown in parentheses: 10, normal (<1.3 ± 0.2 mos.); 9, hind limb muscle weakness (2.1 ± 0.0); 8, wide stance (2.2 ± 0.4 mos.); 7, ataxia (2.5 ± 0.3 mos.); 6, instability with occasional falling (2.9 ± 0.5 mos.); 5, limited walking (3.5 ± 0.5 mos.); 4, able to stand but not walk (3.9 ± 0.5 mos.); 3, cannot stand on 2 consecutive days (humane endpoint, 4.4 ± 0.6 months for untreated SD cats, *n* = 14); 2, lateral recumbency; 1, progressive disease. Also, tremor progression was noted as no tremors, fine tremors, whole-body tremors, hind limb spasticity, or fore limb spasticity.

### Tissue Preparation

Brains were divided into coronal blocks of 0.6  cm from the frontal pole through the cerebellum. Coronal blocks from the right hemisphere were frozen in optimum cutting temperature (OCT) medium and used for analysis of Hex distribution by naphthol staining and Hex-specific activity by 4-methylumbelliferone (4MU) enzyme assays. Coronal blocks from the left hemisphere were halved to 0.3  cm and fixed in 10% formalin or stored at −80℃ for analysis of storage material by high-performance thin layer chromatography (HPTLC).

### Lipid Extraction for HPTLC

CNS tissue was homogenized in 3 ml dH_2_O. An aliquot (100 μl) of each homogenate was set aside for analysis of Hex activity. The remaining homogenate was frozen at −80℃ and lyophilized. The weight of the lyophilized tissue was measured prior to lipid extraction.

Lyophilized tissues were transferred into 50 ml glass screw capped test tubes and rehydrated with 0.5 ml of dH_2_O. Lipids were extracted from the tissue by adding 5 ml CHCl_3_: CH_3_OH 1:1 (v/v). A small magnetic stirring bar was added, and samples were placed on a magnetic stirrer at room temperature overnight (Seyfried et al., 1978; Hauser et al., 2004). Samples were spun at 1200 ×g for 20 min, and the supernatant was collected. Two milliliters of CHCl_3_: CH_3_OH 1:1 (v/v) were added to the pellets prior to spinning a second time. The second supernatant was collected and added to the first; 2.5 ml of chloroform, 8.5 ml of methanol, and 1.6 ml dH_2_0 were added to the supernatant creating a final ratio of 30:60:8 CHCl_3_: CH_3_OH: dH_2_O (v/v/v) (Baek et al., 2010).

### Column Chromatography

Ion exchange chromatography was performed to separate neutral lipids and cholesterol from acidic lipids and gangliosides as previously reported (Macala et al., 1983; Baek et al., 2009). Total lipid extract was applied to a column containing DEAE sephadex (A-25, GE Healthcare). The column was washed twice with 20 ml of solvent A, CHCl_3_: CH_3_OH: dH_2_O 30:60:8 (v/v/v), and the entire neutral lipid fraction consisting of the initial eluent plus washes was collected. This fraction contained the cholesterol, phosphatidylcholine, phosphatidylethanolamine, plasmalogens, sphingomyelin, cerebrosides, and asialo-GM2 (GA2). The column was then washed with 30 ml of solvent B, CHCl_3_: CH_3_OH: 0.8 M NaOAc 30:60:8 (v/v/v) in order to elute and collect acidic lipids and gangliosides.

### Neutral Lipid Purification

Neutral lipids were dried by rotary evaporation and resuspended in 10 ml CHCl_3_:CH_3_OH 1:1 (v/v). To further purify GA2, a 4 ml aliquot of neutral lipids was dried under nitrogen and then base treated with 1 ml of 1 N NaOH at 37℃ for 1.5 hr. The sample was Folch partitioned as previously described (Kasperzyk et al., 2004). The lower phase containing GA2 and cerebrosides was evaporated under nitrogen and resuspended in 4 ml CHCl_3_:CH_3_OH 1:1 (v/v) (Baek et al., 2009).

### Ganglioside and Acidic Phospholipid Purification

The acidic lipid fraction containing gangliosides was dried by rotary evaporation and separated into acidic lipids and gangliosides by Folch partitioning as described previously (Folch et al., 1957; Seyfried et al., 1978; Kasperzyk et al., 2005). After the upper aqueous phase containing gangliosides was transferred, the lower organic phase containing the acidic lipids was dried under nitrogen and resuspended in 10 ml CHCl_3_:CH_3_OH 1:1 (v/v). This fraction contained fatty acids, cardiolipin, phosphatidylserine, phosphatidylinositol, and sulfatides.

### Resorcinol Assay

An aliquot of the upper aqueous phase containing gangliosides was evaporated and analyzed for sialic acid content using a resorcinol assay as previously described (Svennerholm, 1957; Hauser et al., 2004; Kasperzyk et al., 2004). N-acetylneuraminic acid was used as an external standard. Samples were dissolved in 1 ml of resorcinol reagent (40 ml concentrated HCl, 0.125 ml 0.1 M copper sulfate, 5 ml 2% resorcinol stock, brought up to 50 ml with dH_2_O): dH_2_O 1:1 (v/v). Samples were then boiled for 17 min and cooled in an ice bath; 1.5 ml of butyl acetate: 1-butanol 85:15 (v/v) was added to each sample. Samples were vortexed and then centrifuged at 1200 ×g for 1 min. The violet supernatant was removed and analyzed at 580 nm in crystal cuvettes in the Shimadzu UV-1601 UV-visible spectrophotometer (Shimadzu, Kyoto, Japan).

### Base Treatment and Desalting

The remaining gangliosides were evaporated under nitrogen. Dried gangliosides were base treated with 1 ml of 1 N NaOH at 37℃ for 1.5 hr. Salts were removed from gangliosides using a C18 reverse-phase Bond Elute column as previously described (Hauser et al., 2004; Kasperzyk et al., 2004; Baek et al., 2010). The samples were applied to columns that were previously equilibrated with 5 ml each of: CHCl_3_:CH_3_OH 1:1 (v/v), CH_3_OH, and 0.1 M NaCl. The columns were washed slowly with 20 ml dH_2_0 to remove salts. Gangliosides were eluted from the columns using 4 ml CH_3_OH followed by 3 ml CHCl_3_:CH_3_OH 1:1 (v/v) and then dried under nitrogen. Gangliosides were resuspended in 5 ml CHCl_3_: CH_3_OH 2:1 (v/v), and an aliquot was analyzed for sialic acid content.

### Lipid Analysis by HPTLC

All lipids were evaluated qualitatively with HPTLC. Each lane was spotted with 1.5 µg sialic acid for gangliosides, 70 µg of dry weight for neutral lipids, and 200 µg of dry weight for acidic lipids and GA2. Purified lipid standards were purchased from Matreya, Inc. (Pleasant Gap, PA) or were a gift from Dr. Robert Yu (Medical College of Georgia, August, GA). To enhance precision, oleoyl alcohol was added as an internal standard to the neutral and acidic lipid standards and samples. Lipids were spotted on 10 × 20  cm Silica gel HPTLC plates using a Camag Linomat V semiautomatic TLC spotter (Camag Scientific Inc., Wilmington, NC). Gangliosides were developed in a single ascending run for 90 min in CHCl_3_: CH_3_OH: dH_2_O 55:45:10 (v/v/v) containing 0.02% CaCl_2_ (aq). Plates were sprayed with resorcinol-HCl reagent and heated at 95℃ face down for 10 min and then face up for an additional minute in order to visualize gangliosides (Ando et al., 1978; Seyfried et al., 1978). GA2 was developed to the top in a single ascending run in CHCl_3_:CH_3_OH:dH_2_O 65:35:8 (v/v/v) containing 0.22% CaCl_2_ (aq). Plates were sprayed with orcinol-H_2_SO_4_ and heated at 95℃ face up for 5 min to visualize GA2 (Svennerholm, 1957). Neutral and acidic lipid HPTLC plates were developed to a height of 4.5 or 6  cm, respectively, in CHCl_3_:CH_3_OH: CH_3_COOH:HCOOH:dH_2_O 35:15:6:2:1 (v/v/v/v/v). Plates were dried and then developed to the top in C_6_H_14_:C_6_H_14_O:CH_3_COOH 65:35:2 (v/v/v). Lipid bands were visualized by charring with 3% cupric acetate in 8% phosphoric acid solution for 7 min as previously described (Seyfried et al., 1984; Kasperzyk et al., 2004).

### Quantitation of Individual Lipids

The quantitation of individual lipids was performed as previously described (Arthur et al., 2013). Total brain ganglioside distribution was normalized to 100%, and the percentage distribution was used to calculate sialic acid concentration of GM2. To calculate neutral lipids, acidic lipids and GA2 density values were fit to a standard curve of known standard.

### Determination of Lysosomal Enzyme Activity and Distribution

Total Hex activity from samples homogenized for HPTLC was determined as previously described using the synthetic fluorogenic substrate 4-methylumbelliferyl N-acetyl-*β*-D-glucosaminide (MUG; Hauser et al., 2004). For enzyme assays on blocks A–O, several frozen sections (50 µm) were cut from each coronal block, and lysosomal enzyme activity was measured as previously described using synthetic 4-methylumbelliferyl (4MU) fluorogenic substrates (Bradbury et al., 2009): HexA, 4MU-6-sulfo-2-acetamido-2-deoxy-*β*-D-glucopyranoside (MUGS); total Hex, MUG; *α*-mannosidase, 4MU-*α*-D-manopyranoside. Specific activity was expressed as nmol 4MU/mg protein/hr. Histochemical staining for total Hex activity was performed on 40 µm frozen sections using 0.25 mM naphthol AS-BI-N-acetyl-B-D-glucosaminide in the presence of pararosaniline as previously described (Lacorazza and Jendoubi, 1995).

### Statistics

HPTLC and corresponding MUG data were analyzed by one-way analysis of variance (ANOVA) to calculate statistical significance between groups using IMB SPSS Statistics 21 software. For pairwise comparisons of lysosomal enzyme activity, statistics were performed in SAS 9.2 software using the Wilcoxon rank sum test. One-sided testing is reported for directional significance (i.e., significantly higher or lower), which may otherwise not be realized using two-sided testing due to low animal numbers when using a feline model.

## Results

**Figure 1.**
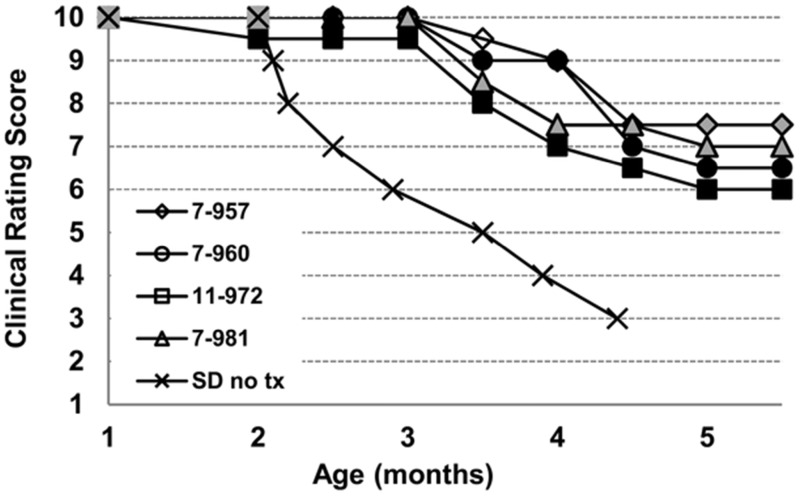
Clinical progression of AAV-treated Sandhoff cats. SD cats from 1 to approximately 5.5 months of age were assigned numerical scores corresponding to discrete stages of clinical disease progression, as defined in the “Materials and Methods” section. Shown are clinical rating scores of four AAV-treated SD cats (7-957, 7-960, 11-972, 7-981) and mean scores for 14 untreated SD cats (SD no tx). The humane endpoint is triggered by a score of 3, which occurs in untreated SD cats at 4.4 ± 0.6 months of age. Treated cats survived in good condition to the predetermined experimental endpoint of approximately 5.5 months of age. Shading of individual symbols represents tremor severity as follows: no shading, no tremor; gray shading, fine tremors; black shading, mild whole-body tremors. Untreated SD cats developed fine tremors at 1.3 (±0.2) months of age and whole-body tremors at 2.4 (± 0.1) months of age. The mean clinical rating scores and standard deviations for treated cats at each age are as follows: 1 mo, 10 ± 0.0; 2 mo, 9.9 ± 0.3; 2.5 mo, 9.9 ± 0.3; 3 mo, 9.9 ± 0.3; 3.5 mo, 8.8 ± 0.6; 4 mo, 8.1 ± 1.0; 4.5 mo, 7.1 ± 0.5; 5 mo, 6.8 ± 0.6; 5.5 mo, 6.8 ± 0.6. Supplemental videos 1 and 2 depict representative untreated and AAV-treated SD cats, respectively. AAV = adeno-associated virus; SD = Sandhoff disease.

Our objective was to determine the degree to which AAVrh8 vectors expressing feline Hex *α* and *β* subunits could correct the biochemical and behavioral symptoms of ganglioside GM2 storage in SD cats when injected into the thalamus combined with intracerebroventricular (Thal/ICV) injections (Bradbury et al., 2013). Clinical disease progression was monitored in animals using a 10-point scoring system based on gait defects in untreated animals (see “Materials and Methods” section for description). Untreated animals experienced hind limb muscle weakness, ataxia, and whole-body tremors by 2.5 (± 0.3) months and progressed to humane end point, defined by inability to stand and a clinical rating score of 3, by 4.4 (± 0.6) months (*n* = 14). Disease progression was delayed in treated animals. At the predetermined end point of 16 weeks posttreatment (approximately five months of age), treated animals received an average score of 6.8 (±0.6) with some animals experiencing mild whole-body tremors while others experienced only fine tremors (Figure 1; Supplemental videos 1 and 2).

**Figure 2.**
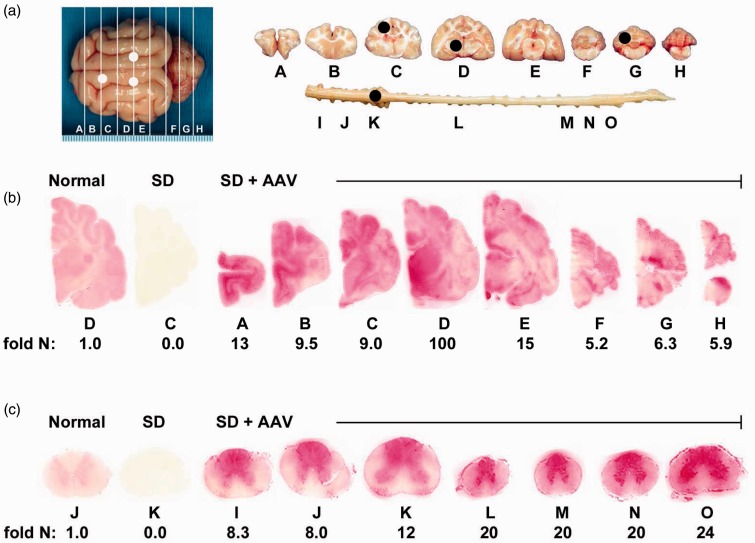
Therapeutic enzyme distribution in the CNS of Sandhoff cats after AAV treatment. SD cats were injected bilaterally in the thalamus and the left lateral ventricle with AAVrh8-fHEXA and AAVrh8-fHEXB (1.1 × 10^12^ vector genomes total), and tissues were collected 16 weeks later. (a) Shown are injection sites (white circles) and 0.6  cm coronal blocks of the brain (A–H) and spinal cord (I–O) collected at necropsy. Blocks were halved and analyzed for enzyme activity (right) or for storage material (left; black circles show biopsy sites for HPTLC). Lysosomal Hex activity (red) detected with naphthol substrate at acidic pH was visualized throughout the brain (b) and spinal cord (c) of a representative, treated SD cat (SD+AAV; 7-960). Corresponding Hex activity against MUGS substrate is shown below each block as fold normal level (fold N). Representative control sections are shown from untreated normal cats along with untreated SD cats, which express ≤0.02 fold normal Hex activity in the brain and spinal cord. The range of specific activities for normal control blocks were brain, 28.1 (G) – 57.4 (D); spinal cord, 8.3 (M) – 17.3 (K) nmol 4MU/mg/hr. AAV = adeno-associated virus; SD = Sandhoff disease.

To determine Hex distribution from the injection site to other regions of the brain, naphthol staining and enzyme assays (MUG and MUGS) were performed for each coronal block and each region of the CNS examined by HPTLC (Figure 2(a)). Histochemical naphthol staining revealed widespread distribution of Hex throughout the brain and spinal cord of treated animals (Figure 2(b) and (c)).

The synthetic substrates MUGS (*α*-subunit preferred) and MUG (for total Hex activity) were used to quantify Hex activity in the CNS, which in untreated SD cats was ≤2% of normal. In AAV-treated SD cats, total Hex activity in brain averaged 3.7 - to 56.6-fold greater in the treated cats than in normal cats, whereas activity in spinal cord was 6.1 - to 16.4-fold greater in treated cats than in normal cats (Table 1). The greatest activity was found in block D, which contains the thalamic injection site. Similar elevations above normal were measured using MUGS substrate (Table 1). Total Hex activity was additionally measured in each brain punch biopsy used for HPTLC analysis and ranged from 2 - to 234-fold normal in the AAV-treated cats (Table 2). Of the sites biopsied for HPTLC, enzyme activity was highest in the thalamic injection site and in the cervical intumescence of the spinal cord.

**Table 1.** Hex Activity in Brain and Spinal Cord of AAV-Treated and Untreated SD Cats.

		Fold normal Hex activity, *Mean* (*SD*)			
Region	Block	Thalamus + ICV (*n* = 4)		SD UnTx (*n* = 5)	
		HexA (MUGS)^a,b^	Hex total (MUG)^a,b^	HexA (MUGS)	Hex total (MUG)
Cerebrum	A	5.0 (5.1)	5.4 (6.7)	0.0 (0.0)	0.0 (0.0)
	B	4.3 (3.5)	3.7 (3.1)	0.0 (0.0)	0.0 (0.0)
	C	23.7(37.7)	24.1 (40.2)	0.0 (0.0)	0.0 (0.0)
	D	72.3 (32.3)	56.6 (16.5)	0.0 (0.0)	0.01(0.01)
	E	9.9 (6.7)	9.7 (7.6)	0.0 (0.0)	0.01 (0.0)
Cerebellum	F	7.1 (3.8)	5.9 (3.9)	0.0 (0.0)	0.01 (0.01)
	G	5.7 (1.3)	5.2 (1.4)	0.01 (0.01)	0.01 (0.01)
	H	5.0 (1.2)	4.5 (1.2)	0.0 (0.0)	0.01 (0.01)
Spinal cord	I	5.3 (2.1)	6.1 (2.5)	0.0 (0.0)	0.0 (0.0)
	J	6.2 (2.4)	6.9 (2.7)	0.0 (0.0)	0.0 (0.0)
	K	8.7 (3.0)	9.6 (3.6)	0.0 (0.01)	0.0 (0.01)
	L	10.9 (7.1)	12.8 (9.0)	0.02 (0.03)	0.01 (0.02)
	M	10.6 (6.3)	10.9 (6.7)	0.02 (0.04)	0.00 (0.00)
	N	12.3 (5.4)	13.3 (6.7)	0.01 (0.02)	0.01 (0.01)
	O	14.7 (6.6)	16.4 (6.6)	0.01 (0.01)	0.0 (0.01)

*Note.* Hex = β-N-acetylhexosaminidase; ICV = intracerebroventricular; MUG = 4-methylumbelliferyl N-acetyl-β-D-glucosaminide; SD = Sandhoff disease.

a Specific activity against the α-subunit-preferred substrate (MUGS) was significantly higher than untreated SD cats in A–H (*p* ≤ .0075 for each block) and I–O (*p* ≤ .0090 for each block). Hex total specific activity against the β-subunit-specific substrate (MUG) was significantly higher than untreated SD cats in A–H (*p* ≤ .01 for each block) and I–O (*p* ≤ .0075 for each block).

b MUGS-specific activity was significantly higher than untreated normal cats (*n* = 4) in A–H and I–O (*p* ≤ .015 for each block). MUG-specific activity was significantly higher than untreated normal cats in A–H and I–O (*p* = .015 for each block).

**Table 2.** Effect of AAV Treatment on Glycosphingolipid Content and β-Hexosaminidase Activity in CNS Regions of the Sandhoff Cat.

Genotype	AAV treatment	*N* ^a^	Hex activity (nmol/mg protein/hr)	Hex activity (fold normal)	Ganglioside content (µg sialic acid/100 mg dry weight)	GM2 (µg/100 mg dry weight)	GA2 (µg/100 mg dry weight)	Cerebrosides (µg/mg dry weight)	Sulfatides (µg/mg dry weight)
**Cortex**									
Normal	–	4	191 ± 32		460 ± 17^††^	ND	ND	64.9 ± 10.2^††^	7.6 ± 1.9^†^
Sandhoff	–	6	7 ± 2	0.04 ± 0.01	1,335 ± 186**	630 ± 99**	1,520 ± 328**	19.0 ± 7.1**	2.5 ± 0.5*
Sandhoff	Thal/ICV^b^	3	408 ± 171^†^	2.14 ± 0.9^†^	507 ± 45^†^	37 ± 14^††^	41 ± 41^†^	31.0 ± 2.3*	3.9 ± 0.2
**Cerebellum**									
Normal	–	4	179 ± 39		273 ± 32^†^	ND	ND	67.0 ± 7.7^†^	16.3 ± 2.2^†^
Sandhoff	–	7	6 ± 1	0.03 ± 0.01	532 ± 61*	251 ± 39**	1,533 ± 286**	36.3 ± 21*	9.1 ± 0.9**
Sandhoff	Thal/ICV^b^	3	1,272 ± 305**^††^	7.11 ± 1.70**^††^	330 ± 12^†^	21 ± 12^††^	45 ± 45^††^	29.0 ± 3.0*	6.2 ± 0.7**
**Thalamus**									
Normal	–	4	220 ± 39^†^		314 ± 24^††^	ND	ND	48.1 ± 10.7^†^	22.2 ± 3.9^††^
Sandhoff	–	6	7 ± 1*	0.03 ± 0.00*	1,253 ± 135**	446 ± 50**	1,876 ± 192**	18.4 ± 4.4*	5.7 ± 1.2**
Sandhoff	Thal/ICV^b^	3	51,450 ± 15,191**^††^	233.86 ± 69.05**^††^	473 ± 5^††^	9 ± 6^††^	ND	47.6 ± 2.8^†^	12.3 ± 3.4*
**Cervical Intumescence**									
Normal	–	4	392 ± 143		89 ± 3^††^	1 ± 1^††^	19 ± 19**	65.4 ± 20.1	13.9 ± 3.1
Sandhoff	–	6	5 ± 3	0.01 ± 0.01	309 ± 42**	126 ± 24**	324 ± 61^††^	49.2 ± 14.1	16.3 ± 4.0
Sandhoff	Thal/ICV^b^	3	2,950 ± 319*^†^	7.53 ± 0.81*^†^	104 ± 14^††^	2 ± 2^††^	ND	28.2 ± 11.3	8.6 ± 3.9

*Note*. Values are expressed as the mean ± standard error of the mean (*SEM*). Values are significantly different from the normal *p ≤ .05 and **p ≤ .001. Values are significantly different from the nontreated Sandhoff ^†^ *p* ≤ .05 and^ ††^ *p* ≤ .001. AAV = adeno-associated virus; CNS = central nervous system; Hex = β-N-acetylhexosaminidase; ICV = intracerebroventricular; GA2 = Asialo-GM2.

a *N* is the number of independent samples analyzed.

b Cats received injections in the thalamus and the lateral ventricle.

Thal/ICV AAV treatment reduced total ganglioside content by 96%, 89%, 87%, and 95% in cortex, cerebellum, thalamus, and spinal cord regions, respectively, to levels comparable with those found in normal animals (Table 2).

**Figure 3.**
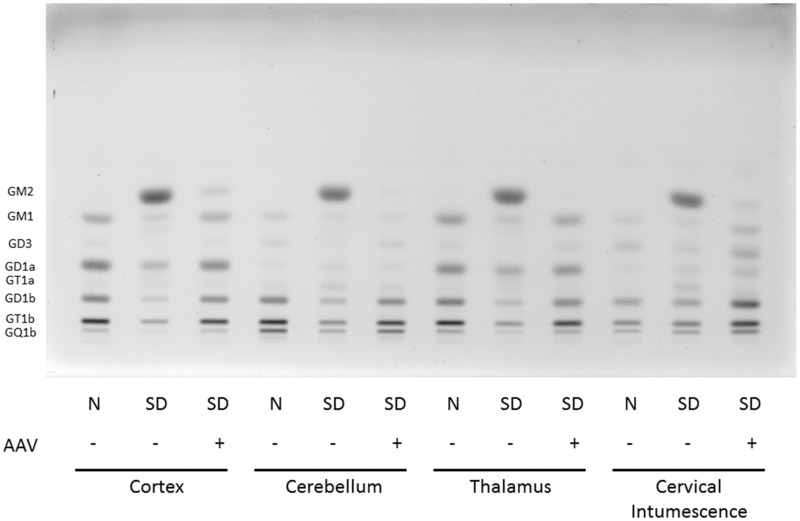
HPTLC of gangliosides from Sandhoff cat cortex, cerebellum, thalamus, and spinal cord (cervical intumescence). Gangliosides from normal cats (N), Sandhoff disease cats (SD), and Sandhoff disease cats treated with AAV vectors were separated in a single ascending run with CHCl_3_:CH_3_OH;dH_2_0, 55:45:10 (v/v/v) with 0.02% CaCl_2_. 1.5µg of sialic acid was spotted for each sample. The bands were visualized with the resorcinol-HCl spray. Ganglioside positions are shown on the left-hand side of the plate. AAV = adeno-associated virus; SD = Sandhoff disease; HPTLC = high-performance thin layer chromatography.

GM2 accounted for approximately 40% of the total ganglioside in the various CNS regions of untreated cats. Treatment with Thal/ICV AAV injections resulted in a ≥92% decrease of GM2 storage in all CNS regions tested with a 98% decrease in the thalamus (Figure 3). Correction of GM2 storage varied for each animal tested as well as between regions for each individual cat. In some animals, AAV treatment decreased GM2 concentration to below detectable levels, as in the normal cats.

Similar reductions were documented in the levels of asialo GM2 (GA2) in AAV-treated cats. GA2 was undetectable in the CNS regions examined in normal cats but was a major storage material in SD cats. AAV treatment reduced GA2 to undetectable levels in the thalamus and cervical intumescence of all SD cats (Table 2), while average reduction was ∼ 97% in the cortex and cerebellum biopsy sites. Two Thal/ICV treated animals had no detectable GA2 accumulation in any of the regions tested (data not shown for individual animals) while the remaining cats varied in the amount of correction in each region.

**Figure 4.**
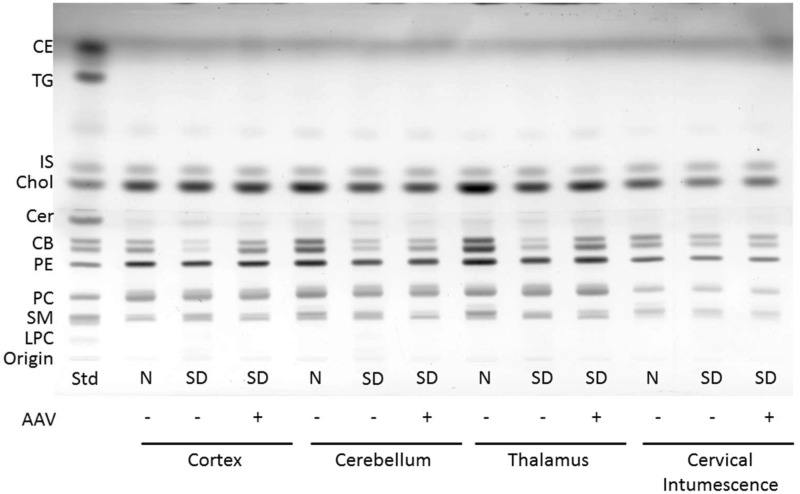
HPTLC of neutral lipids from Sandhoff cat cortex, cerebellum, thalamus, and cervical intumescence. Sample abbreviations are as listed in Figure 3. Std, standard. The plate was developed to a height of 4.5  cm with CHCl_3_:CH_3_OH: CH_3_COOH:CHOOH:dH_2_O 35:15:6:2:1 (v/v/v/v/v) and then developed to the top with C_6_H_14_:C_6_H_14_O:CH_3_COOH 65:35:2 (v/v/v). The amount of neutral lipid spotted per lane was equivalent to 70 µg tissue dry weight. The bands were visualized by charring with 3% cupric acetate in 8% phosphoric acid solution. CE = cholesterol esters; TG = triglycerides; IS = internal standard (oleyl alcohol); Chol = cholesterol; Cer = ceramide; CB = cerebrosides (doublet); PE = phosphatidylethanolamine; PC = phosphatidylcholine; SM = sphingomyelin; LPC = lysophosphatidylcholine; AAV = adeno-associated virus; SD = Sandhoff disease; HPTLC = high-performance thin layer chromatography.

**Figure 5.**
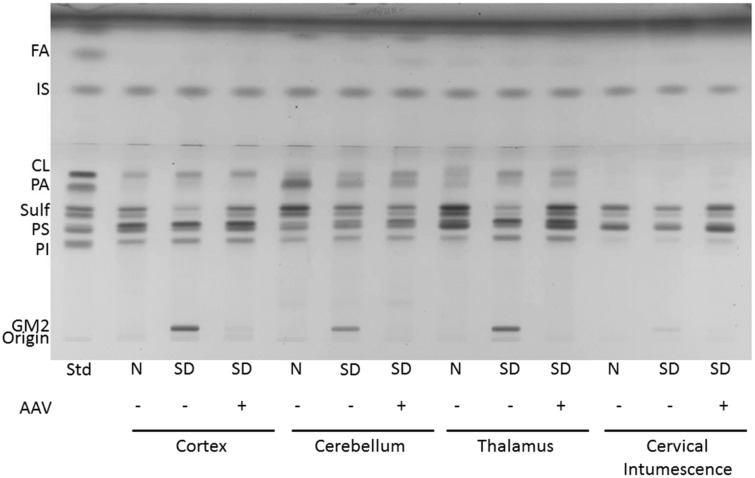
HPTLC of the acidic lipids from Sandhoff cat cortex, cerebellum, thalamus, and cervical intumescence. Sample abbreviations are as listed in Figure 3. Std, standard. The plate was developed to a height of 6  cm with CHCl_3_:CH_3_OH: CH_3_COOH:CHOOH:dH_2_O 35:15:6:2:1 (v/v/v/v/v) and then developed to the top with C_6_H_14_:C_6_H_14_O:CH_3_COOH 65:35:2 (v/v/v). The amount of acidic lipid spotted per lane was equivalent to 200 µg tissue dry weight. The bands were visualized by charring with 3% cupric acetate in 8% phosphoric acid solution. FA = fatty acids; IS = internal standard (oleyl alcohol); CL = cardiolipin; PA = phosphatidic acid; Sulf = sulfatides (doublet); PS = phosphatidylserine; PI = phosphatidylinositol.

The concentration of cerebrosides and sulfatides was significantly lower in the brain regions of untreated SD cats compared with normal cats (Table 2, Figures 4 and 5). This is consistent with previous reports of lower myelin-enriched glycolipids in SD mice, cats, and humans (Sandhoff et al., 1971; Baek et al., 2009). Treatment normalized the level of cerebrosides and partially restored sulfatides in the thalamus but was insufficient to restore normal levels of these myelin-associated lipids in the cortex or cerebellum (Table 2). No differences were found for the concentration of cerebrosides or sulfatides in the cervical spinal cord between normal and SD cats regardless of treatment. However, unusually high variability in all cohorts may have contributed to inconclusive findings from this region.

**Figure 6.**
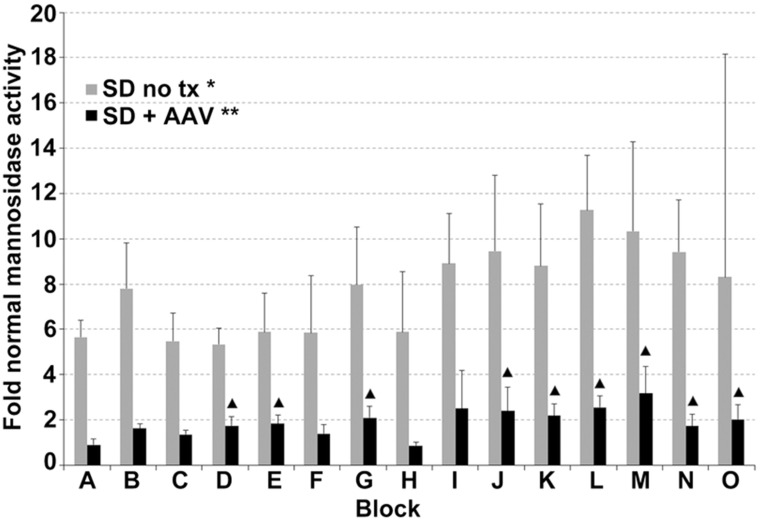
Normalization of lysosomal α-mannosidase activity in the CNS of SD cats 16 weeks posttreatment. Lettering of brain and spinal cord blocks correspond to Figure 2. Error bars represent standard deviation. *, all samples from untreated SD cats were significantly higher than normal (*p* = .010 for each block); **, all samples from treated SD cats were significantly lower than untreated (*p* ≤ .019 for each brain block; *p* ≤ .037 for each spinal cord block); ▴, samples from treated cats that were significantly higher than normal (*p* ≤ .030). AAV = adeno-associated virus; SD = Sandhoff disease; CNS = central nervous system.

Elevation of nonmutant lysosomal hydrolases often occurs in lysosomal storage diseases (McCurdy et al., 2014). CNS *α*-mannosidase activity was 5.6 - to 11.3-fold higher in untreated SD cats than in normal cats (*p* = 0.01 for each block; Figure 6). AAV treatment significantly decreased mannosidase activity in all CNS regions, though levels remained significantly above normal in three of eight brain regions and six of seven spinal cord blocks.

## Discussion

No effective treatments presently exist for persons with SD. Recent studies in murine and feline models have shown the potential for AAV treatment in the management of ganglioside storage diseases (Cachon-Gonzalez et al., 2006; Broekman et al., 2007; Baek et al., 2010; Sargeant et al., 2011; Cachon-Gonzalez et al., 2012; Bradbury et al., 2013; McCurdy et al., 2014). Here, we examined the effects of thalamic injections, in combination with ICV injection, on disease progression of SD cats using AAVrh8 vectors expressing feline Hex *α* and *β*. Hex activity and lipid concentrations were evaluated in different regions of the SD cat CNS. Previous studies showed that axonal transport could facilitate delivery of lysosomal enzymes throughout the CNS (Passini et al., 2002; Broekman et al., 2009). The highly interconnected thalamus was chosen as the site for AAV injection to maximize the distribution of Hex from vector-transduced cells (Baek et al., 2010; Baxter, 2013; Constantinople and Bruno, 2013). However, previous work (Bradbury et al., 2013) demonstrated that thalamic injection alone was not sufficient to restore Hex activity to the cerebellum. Because of the vascularity of the posterior fossa and the surgical risk associated with direct injection of the cerebellum, we selected ICV injections for cerebrospinal fluid (CSF)-based treatment of the cerebellum, and potentially the spinal cord. Enzyme activity in all regions of the treated CNS was significantly increased compared with both untreated SD animals and normal animals, and Hex reached 5 - to 7-fold normal in the cerebellum compared with a maximum of 0.4-fold normal in animals treated by thalamic injection only (Bradbury et al., 2013). Inclusion of ICV injection or a similar route is required for effective treatment of the cerebellum. The highest level of enzymatic activity occurred in the thalamic injection site, where Hex activity was 234-fold above normal. While other studies using AAVs have reported brain toxicity caused by overexpression of proteins (Georgievska et al., 2002; Klein et al., 2006), our previous study using this vector found no evidence of neuron loss or clinical neurological symptoms in normal cats >1.5 years postinjection indicating no brain toxicity as a result of AAV-mediated overexpression of Hex (Bradbury et al., 2013). For this reason, we do not believe that the high levels of enzyme cause pervasive cytotoxicity in the cat CNS.

In addition to axonal transport of Hex to areas of the CNS remote from the injection site, the therapeutic mechanism in our study is likely to involve transmission of the vector and enzyme through the perivascular space of Virchow–Robin (Broekman et al., 2009; Cachon-Gonzalez et al., 2012). Also, it is known that AAV vectors transduce ependymal cells (Yamazaki et al., 2014; Yang et al., 2014) and that lysosomal enzyme is distributed throughout the brain parenchyma after CSF-mediated delivery of AAV (Liu et al., 2005; Haurigot et al., 2013). However, because cats in the current study were treated by both CSF and thalamic injection, we cannot strictly determine the contribution of each individual route to Hex activity in the brain parenchyma.

The storage of GM2 and GA2 in SD causes lysosomal and neuronal swelling (Cork et al., 1977, 1978), cellular apoptosis, and CNS inflammation (Huang et al., 1997; Jeyakumar et al., 2003; Sargeant et al., 2011). To prevent pathology, SD treatment should ultimately correct the underlying defect by decreasing lipid storage in lysosomes. Treatment with AAV decreased GM2 storage, as well as GA2 storage, by greater than 92% in all regions tested. Though ganglioside levels in SD cats after treatment were not statistically different from normal, slight elevations may have been caused by uneven Hex distribution, so that not all cells had sufficient activity for complete clearance of storage material. As seen in Figure 2, our treatment approach generated regions of intense activity that tapered to much lower levels within the same coronal section. Nevertheless, neuronal morphology was normalized almost completely throughout the brain (data not shown). Discrete areas of persistent neuropathology were thought to coincide with areas that received the lowest levels of Hex activity. Rare areas of perivascular cuffing and cellular infiltrate were present in the cerebral cortex of treated SD cats but were much less pervasive than in our previous study in which mouse Hex was used for treatment (Bradbury et al., 2013).

Although gangliosides are mostly stored in CNS gray matter, histopathologic changes have been seen in white matter of mice, cats, and humans with SD (Koelfen et al., 1994; Kroll et al., 1995; Baek et al., 2009). The myelin-associated lipids, cerebrosides and sulfatides, are also decreased in mice, cats, and humans with SD (Sandhoff et al., 1971; Baek et al., 2009), suggesting that dysmyelination contributes to SD pathogenesis (Kroll et al., 1995). Our results showed that AAV treatment fully or partially restored the level of cerebrosides and sulfatides in the thalamus. The lack of correction in the cortex and cerebellum despite reduced ganglioside storage requires further investigation but may improve at time points beyond 16 weeks postinjection. If cerebroside/sulfatide defects persist with time, it is possible that uneven Hex distribution in the brain prevents full correction of the disease phenotype in all cells. Alternatively, strong evidence has been reported that an early myelin defect in SD mice is refractory to standard gene therapy (Cachon-Gonzalez et al., 2014), which could explain the lack of cerebroside/sulfatide restoration distal to the injection site in the current study. Focal rather than global normalization of myelin defects may be responsible for the incomplete arrest of disease progression, though the disease phenotype was dramatically improved by treatment.

The differences we found for total ganglioside concentration between the various regions of the untreated cat brain are consistent with observations from previous studies (Ueno et al., 1978; Byrne and Ledeen, 1983; Baek et al., 2008) and from human SD patients (Baek et al., 2009). Regardless of the concentration of storage material across different sample locations of untreated animals, total ganglioside content was significantly reduced in all CNS regions after AAV treatment. When individual gangliosides GM2 and GA2 were quantified, some variability was noted between treated animals, though significant correction was seen in each region, and every animal had substantial clearance of ganglioside.

Our study shows the effectiveness of AAV gene therapy in restoring Hex activity to normal or above-normal levels and attenuating the major lipid abnormalities in SD cats. While the initial findings are unusually promising for a large animal model of lysosomal disease, further studies are needed to evaluate the long-term effects of the treatment. Examination of clinical effect, enzyme activity, and lipid concentrations of animals that are followed long-term will allow us to better assess the potential for translation into human treatment.
